# A single experience in the conduction of clinical trial during COronaVIrusDisease-2019 pandemic

**DOI:** 10.37349/etat.2023.00168

**Published:** 2023-09-07

**Authors:** Zelmira Ballatore, Amalia Goudas, Francesco Bozzi, Alessandra Lucarelli, Michela Burattini, Giulia Ricci, Francesco Savino, Rossana Berardi

**Affiliations:** Regina Elena Cancer Institute, Italy; ^1^Department of Medical Oncology, AOU della Marche, 60126 Ancona, Italy; ^2^Medical Oncology, Università Politecnica delle Marche, 60126 Ancona, Italy

**Keywords:** COronaVIrusDisease-2019, clinical trial, pandemic, cancer care

## Abstract

**Aim:**

From the start of the pandemic, several aspects of healthcare policies changed, not least the clinical trials management from recruiting capabilities to the protocol compliance in terms of schedule of procedures, follow-up visits, staff constraints and monitoring. This study aims to assess the impact of the COronaVIrusDisease-2019 (COVID-19) pandemic in the conduction of clinical trials at the site of clinical oncology, Ancona (Italy), to identify the strengths and weaknesses upfront the past emergency, and to select better strategies for future similar situations.

**Methods:**

Data from February to July of the years 2019, 2020 and 2021 were collected and three practical parameters of the trial unit were investigated: milestones, performance, and impact.

**Results:**

The trials mean numbers were 18, 24, and 23, in 2019, 2020, and 2021 respectively. The pre-Site Initiation Visit (PRE-SIV) rate grew from 66.6% in 2019 to 95.5% in 2021 with a deflection in 2020. Protocol deviations were 40 in the period February-July 2019, in the same period of 2020 the number of deviations increased due to COVID related ones, then there was a significant total decrease in February-July 2021. In 2020 and 2021, all the investigator meetings were online.

**Conclusions:**

The growing number of remote Site Initiation Visit (SIV) and meetings over the last 3 years suggests the feasibility of the on-line processes. The significant reduction in protocol deviations during 2021 is probably due to an under check of data during a pandemic. But that is also a possible key indicator of the coping strategy made out by clinical oncology to guarantee the continuity of care in clinical trials and to offer new opportunities of cancer care in a bad scenario such as a pandemic one.

## Introduction

Clinical trials are the highest contribution to improve knowledge and progress in various settings of medicine. In addition, they add great opportunities for cancer care offering new treatments and benefiting from advances in scientific research [1].

COronaVIrusDisease-2019 (COVID-19) globally spread from February 2020, becoming pandemic and leading to a new scenario with the reorganization of medical assistance and medical research too [2].

From the start of the emergency, substantial changes in public policies have impacted on travel, public meetings and access to care in hospital and outpatient settings, disrupting aspects of clinical care and involving management of clinical studies [3].

Health measures have been adopted to deal with the virus by strengthening hospital intensive care wards for COVID-19 patients, activating temporary areas to avoid crowding [4].

The Italian hospital and oncology department were involved in dealing with the emergency and the reorganization defining treatment and instrumental priorities. During the pandemic, it was necessary to balance between risk and benefit of hospital admission considering the probability of contagious severe acute respiratory syndrome coronavirus 2 (SARS-CoV2) within hospitals, but at the same time to continue with life saving therapy [5].

Many clinical trials immediately suspended the accrual, and a number of biopharmaceutical companies announced delays in their trial plans and site activation in all the countries [6].

Meditata conducted a survey all over the world (Europe/United Kingdom, Middle-East Africa, United States of America, Asia) on the impact of COVID pandemic over clinical trials. For 69% responders, COVID-19 affected their ability to conduct ongoing trials and for 78% of responders COVID-19 had a negative influence on the beginning of new trials [7].

There have been significant changes in the trials conduction, from the enrollment, to the compliance to the protocol, the respect of the schedule visits, staff constraints and monitoring [8].

More than four thousand experimental studies were active in Italy at the end of January 2020 [9].

However, considering the rate of new patients in clinical trials in Italy, between March 2020 and March 2019 there was a reduction of 53% in enrollment [10].

To deal with the pandemic scenario, regulatory entities [European Medicines Agency (EMA) and Italian Medicines Agency (AIFA)] have drawn up guidelines in order to reorganize clinical trials and to respect the standards of good clinical practice [11, 12].

The main national and international scientific societies, as the Italian Association of Medical Oncology (AIOM), the European Society for Medical Oncology (ESMO) and the American Society of Clinical Oncology (ASCO) contributed to the draft of the guidelines [13–15].

Guidelines address aspects of clinical trial management, such as protocol modification, communications with the institutional review board/ethics committee, investigational product distribution, remote site monitoring, informed consent process and the importance of maintaining data integrity and the audit trail [16].

The aims of this study were to evaluate the impact of the COVID-19 pandemic in the interventional clinical trials at the clinical oncology of the University Hospital of Marche, in Ancona (Italy) and to identify the strengths and the weaknesses adopted upfront the past emergency.

## Materials and methods

All data concerning interventional and active trials (profit and non-profit) were collected, from phase I to phase III, conducted from February to July of the years 2019, 2020 and 2021.

The following areas were identified:

(1). Milestones.
(2). Performance.
(3). Impact.

The milestones data included:

(1). Number of recruiting trial.
(2). Number of pre-Site initiation visit (PRE-SIV), Site Initiation Visit (SIV), Close Out Visit (COV) of the site.
(3). Monitoring visit.
(4). Investigator meeting.
(5). First patient in/last.

For each trial, the following information was collected:

(1). State of the trial (active/suspended/closed).
(2). Protocol acronym, sponsor and phase.
(3). Setting of disease.
(4). Setting of population.
(5). Treatment arms.
(6). PRE-SIV date.
(7). SIV date.
(8). Monitoring visit date and number.
(9). COV date.
(10). Investigator’s meeting date and number.
(11). List of patients enrolled in each trial with the date of screening, eligibility of the patient (enrolled or screening failure), status of the patient in relation to the treatment of the study (treatment ongoing/off study, follow-up, death).

The performance key indicators included:

(1). Enrollment rate in active trials.
(2). Compliance with the schedule visits.
(3). Patient safety based on the number of protocol deviations. A protocol deviation is any change from the study design under the investigator’s control and not approved by the ethics board. While, COVID-19 related protocol deviation is a protocol deviation due to the pandemic impact on the organization, management, patient safety or other.

The source documents used to collect all the above information were:

(1). Follow-up letters of the monitoring visits sent to the site by the contract research associate (CRA).
(2). Annual report (follow up form) sent to the ethics committee by the site for each trial.
(3). Case report form (CRF).
(4). Paper medical records.
(5). Protocols and patients database.
(6). Software human including work agenda and visit planning.

### Impact

About the semesters of the three considered years, the following data related to the trial enrollment status were taken into consideration:

(1). Enrollment suspended: study temporarily interrupted and then restarted.
(2). Enrollment withdrawn: study permanently suspended and not restarted.
(3). Enrollment terminated: study concluded for the achievement of target/study objective.

All data was collected and processed with the Microsoft Excel program (version 2019 16.0.6742.2048).

## Results

All interventional recruiting studies in the considered periods were included.

The average number of interventional recruiting studies was 18 studies in 2019, 24 in 2020 and 23 in 2021. The maximum number of recruiting studies was 21 in July 2019, 28 in February of 2020 and to 25 in July of 2021.

### Milestones

**Figure 1:**
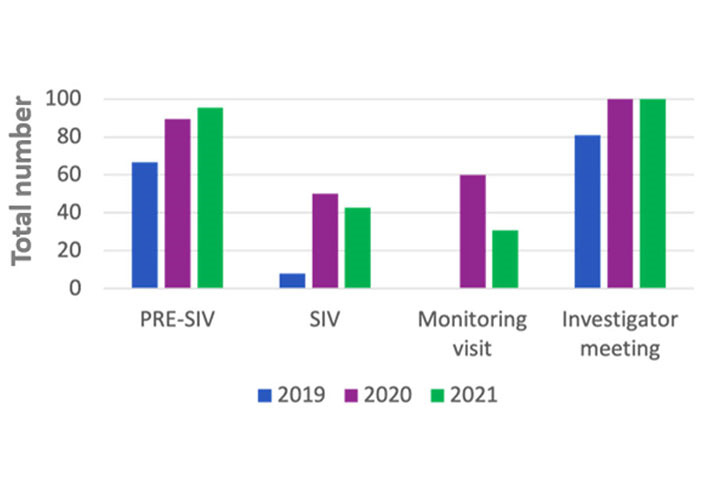
Milestone remotely performed during the three different years

In the 2019 the rate of PRE-SIV remotely conducted were 66.6%, while 33.3% were on site, in the 2020 the rate of remote were 89.5% remotely and 10.5% on site, in the 2021 almost all the PRE-SIV were conducted remotely (Table 1 and Figure 1).

In the 2019 the rate of SIV remotely performed was one (7.7%), while 12 (92.3%) were on site, in the 2020 half of them were on site and half were remote, in the 2021, the rate of remote were 6 (42.8%), the remnant 8 (57.2%) were on site (Table 1 and Figure 1).

In 2019 all the monitoring visits carried out were on site (83). In the 2020 the rate of monitoring visits remotely conducted were 60.3% (35), while 39.7% (23) on site, in the 2021 the rate remotely was 30.9% (21) remotely and 69.1% (47) on site (Table 1).

In March and April 2019, the monitoring visits were on site for a total number of 12 and 16 respectively, in March and April 2020 were all remotely and 8 and 6, respectively (Table 1).

In the 2019 the rate of investigator meetings remotely conducted were 17 (81%) and 4 (19%) were on site, in the 2020 and in the 2021 all investigator meetings were remote (14 and 23 respectively) (Table 1 and Figure 1).

### Performance

**Figure 2:**
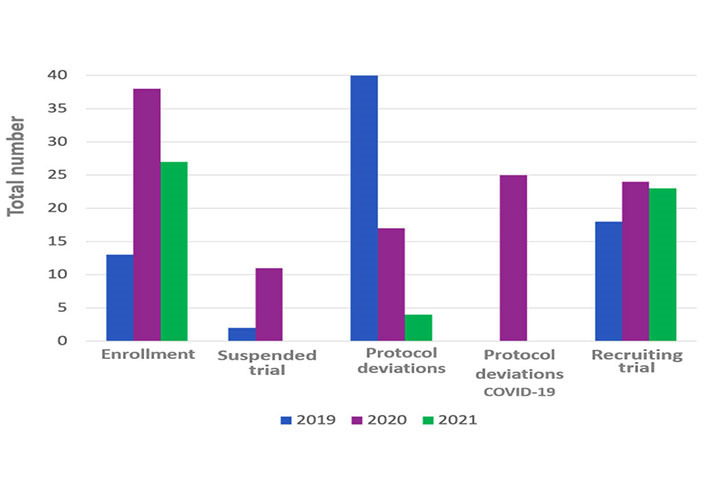
Performance and impact indicators during the three different years

In the 2019 the total number protocol deviations were 40, in the 2020 they were 42 and in the 2021 were 4. About the protocol deviations carried out during 2020, 17 out 42 related to COVID-19 pandemic (Table 2 and Figure 2).

In the considered period during 2019, the enrolled patients were 13, in 2020, they were 38 and in 2021, the total was 27 (Table 2).

In July 2019 there was the maximum number of patient visits (63 visits each months), while during 2020 the maximum number was in February and April (57 visits each months), in 2021 it was during March (51 visits each months) (Table 2 and Figure 2).

### Impact

In the 2019 from February to July the range of active studies was 18, whereas the suspended trials were 2 and the terminated trials were 5 (Figure 2).

In 2020, the active studies were 24, whereas the suspended trials were eleven and terminated trials were 6. In the 2021, the active trials were 23, while there was no suspended trial and the terminated ones were 6 (Table 3).

## Discussion

The pandemic has a global impact on cancer care and clinical trials have been one of the most affected areas [17].

Results of a systematic review by Sathian et al. [10] showed how two-thirds of the respondents stopped subject enrollment in ongoing clinical trials, one-third interrupted the randomization process and fifty percent of respondents delayed or planned to delay the activation of new studies.

Looking at the data, it’s evident how the pandemic led to a modification of study site selection and to the conduction of clinical trials, also. A major limitation of this study stands in the comparison of data from 2019 to 2021, not considering useful previous data due to an internal reorganization during 2018.

The number of PRE-SIVs shows an increase rate over time despite the pandemic, but the performance has changed. In fact, the percentage of PRE-SIV remotely conducted grew from 66.6% in 2019 to 95.5% in 2021.

In the meanwhile, there was a clear reduction in the number of SIVs during 2020 compared to 2019 and 2021. This decrease occurred during March and April 2020. Literature data confirmed similar results in other countries in which 85% of the trial sites postpone SIV during pandemic [18].

The number of remote SIV growth over the last 3 years from 7.8% to 42.8%, suggesting the feasibility of the telematics process. Literature data confirms that over 70% of sites organized SIV and monitoring visits on-line, reducing on site visits for sponsors and promoting easier approval process and improving the cost effectiveness of clinical trials [8, 19].

In 2020, the participating investigator meetings were less than those conducted in 2019, and in the 2020 and 2021 the meetings were 100% remotely, bringing a huge change.

Despite the reduction in milestones in 2020 due to the suspended trials, data showed that COVID-19 has brought a new work modality at site. In fact, Webex became a reality at work in 2020, they were confirmed in 2021 and they are still in use now.

According to literature data, after a sharp early decline, the enrollment of new participants and ongoing study visits recovered during the COVID-19 pandemic. This recovery was accompanied by the increased use of electronic tools and new approaches such as remote visits and telemedicine [20].

So, even if there were a reduction in the number of PRE-SIV, SIV, monitoring visits, the fewer of those have been carried out with a new modality taking advantage of the technologies.

Regulators issued guidance published on statistical mitigation approaches, while sponsors applied telemedicine and remote patients to avoid limited data gaps and protocol deviations [21].

An important number of deviations due to the pandemic were highlighted in Ancona. These findings are consistent with other studies conducted in other countries in which about 50–60% of the protocol deviations were COVID-19 related [22].

Interestingly, in 2021 the protocol deviations were fewer, but this was probably due to the reduction of monitoring visits, and because the source data checks were not carried out properly. But a probable improvement of trial performance was also due to the site capabilities of coping with pandemic impact, even if there were a high number of active trials.

Performance items showed an increase in patient enrollment in 2020 compared to 2019. This finding is site specific and justifiable by the fact that during 2019, there were more PRE-SIV and SIV leading to the opportunity of a high number of active studies in 2020, while in 2021 there are fewer enrolled patients due to the reduction of PRE-SIV and SIV during 2020.

By the analysis of the contract research organization IQVIA and by the interrogation of ClinicalTrials.gov, only 14% of institutions continued with active enrollment during the pandemic and the oncology clinic in Ancona was one of those. There was an increase in enrollment during 2020, despite the restrictions related to the pandemic and becoming one of the clinical sites that have been able to face pandemic maintaining the performance [16].

In conclusion, during the pandemic the oncology clinic was able to guarantee the continuity of care of clinical trials patients and to offer new experimental opportunities carrying out the prevention of SARS-CoV2 infections.

## Abbreviations

COVID-19: COronaVIrusDisease-2019
PRE-SIV: pre-Site Initiation Visit
SIV: Site Initiation Visit

## Declarations

### Availability of data and materials

The data analyzed in this study was obtained from [Internal database, trial report, newsletters of clinical study and internal agenda]. Requests for access to these datasets should be directed to (Zelmira Ballatore, zelmira.ballatore@gmail.com).

### Copyright

© The Author(s) 2023.
